# Transport systems, intracellular traffic of intermediates and secretion of β-lactam antibiotics in fungi

**DOI:** 10.1186/s40694-020-00096-y

**Published:** 2020-04-25

**Authors:** Juan F. Martín

**Affiliations:** Área de Microbiología, Departamento de Biología Molecular, Universidad de León, León, Spain

**Keywords:** Secondary metabolites, Penicillins, Cephalosporins, ACV, LLD α-aminoadipyl-cysteinyl-valine, IPN, isopenicillin N, Peroxisomes, Antibiotics secretion, Intracellular traffic, MFS transporters, Subcellular compartmentalization

## Abstract

Fungal secondary metabolites are synthesized by complex biosynthetic pathways catalized by enzymes located in different subcellular compartments, thus requiring traffic of precursors and intermediates between them. The β-lactam antibiotics penicillin and cephalosporin C serve as an excellent model to understand the molecular mechanisms that control the subcellular localization of secondary metabolites biosynthetic enzymes. Optimal functioning of the β-lactam biosynthetic enzymes relies on a sophisticated temporal and spatial organization of the enzymes, the intermediates and the final products. The first and second enzymes of the penicillin pathway, ACV synthetase and IPN synthase, in *Penicillium chrysogenum* and *Aspergillus nidulans* are cytosolic. In contrast, the last two enzymes of the penicillin pathway, phenylacetyl-CoA ligase and isopenicillin N acyltransferase, are located in peroxisomes working as a tandem at their optimal pH that coincides with the peroxisomes pH. Two MFS transporters, PenM and PaaT have been found to be involved in the import of the intermediates isopenicillin N and phenylacetic acid, respectively, into peroxisomes. Similar compartmentalization of intermediates occurs in *Acremonium chrysogenum;* two enzymes isopenicillin N-CoA ligase and isopenicillin N-CoA epimerase, that catalyse the conversion of isopenicillin N in penicillin N, are located in peroxisomes. Two genes encoding MFS transporters, *cefP* and *cefM,* are located in the early cephalosporin gene cluster. These transporters have been localized in peroxisomes by confocal fluorescence microscopy. A third gene of *A. chrysogenum*, *cefT*, encodes an MFS protein, located in the cell membrane involved in the secretion of cephalosporin C, although *cefT*-disrupted mutants are still able to export cephalosporin by redundant transporters. The secretion of penicillin from peroxisomes to the extracellular medium is still unclear. Attempts have been made to identify a gene encoding the penicillin secretion protein among the 48 ABC-transporters of *P. chrysogenum*. The highly efficient secretion system that exports penicillin against a concentration gradient may involve active penicillin extrusion systems mediated by vesicles that fuse to the cell membrane. However, there is no correlation of pexophagy with penicillin or cephalosporin formation since inactivation of pexophagy leads to increased penicillin or cephalosporin biosynthesis due to preservation of peroxisomes. The penicillin biosynthesis finding shows that in order to increase biosynthesis of novel secondary metabolites it is essential to adequately target enzymes to organelles.

## Introduction

Filamentous fungi, plants and some bacteria are known to produce thousands of specialized (secondary) metabolites [20] that have an important impact on the human society [44]. Some of them have beneficial effects [21] as antibiotics, antitumor and anticholesterolemic agents and others are potent toxins that may produce important diseases in humans and animals [118]. In addition, some fungal secondary metabolites protect the producing strains against fungivors [15, 90]. Secondary metabolites are classified in several broad classes that include polyketides (PK), non-ribosomal peptides (NRP), terpenes, ribosomal synthesized modified peptides, NRP-PK hybrid compounds and other complex heterocyclic secondary metabolites [47, 63]. Impressive progress has been made in recent years on the characterization of secondary metabolites biosynthetic enzymes and the molecular genetics of the encoding gene clusters using the omics tools [117]. Advances have also been made on our knowledge of the nutritional and environmental stress sensing systems and signal transduction cascades in fungi [77]. This article is focused in a particular class of non-ribosomal peptides that are converted to β-lactam antibiotics in fungi. The molecular genetics of biosynthesis of penicillin and cephalosporins serves as an excellent model to understand the mechanisms of transport and secretion of other secondary metabolites.

## Temporal organization of expression of secondary metabolites biosynthetic genes

Expression of genes encoding secondary metabolites in gene clusters is frequently coregulate [35, 63] what favours the formation of equimolecular amounts of the different biosynthetic enzymes [46] and also of the correct amounts of regulatory proteins encoded by genes that are frequently, although not always, situated in the corresponding gene clusters [36]. In the last decades significant evidence on the temporal organization of expression of different gene in a given gene cluster and also of subcellular localization of the biosynthetic enzymes has been published [52, 63, 73, 74]. Even before the arrival of the omics era several examples of sequential formation of intermediates and final products of a biosynthetic pathway were described. Time-dependent formation of the intermediate isopenicillin N during penicillin G production in *P. chrysogenum* cultures was observed in early reports by Revilla et al. [87] and also in the formation of isopenicillin N/penicillin N and its late conversion to cephalosporin C in *Acremonium chrysogenum* [116].

The molecular basis of this sequential biosynthesis of intermediates and final products suggests that this is due to limitation of a key nutrient, either phosphate, carbon or nitrogen source, whereas the conversion of middle and late intermediates in the final products of the pathway is delayed considerably until the genes for the conversion of intermediates in the pathway are expressed [68].

## The biosynthesis of β-lactam antibiotics: a model for the compartmentalization and transport of secondary metabolites in fungi

**Fig. 1:**
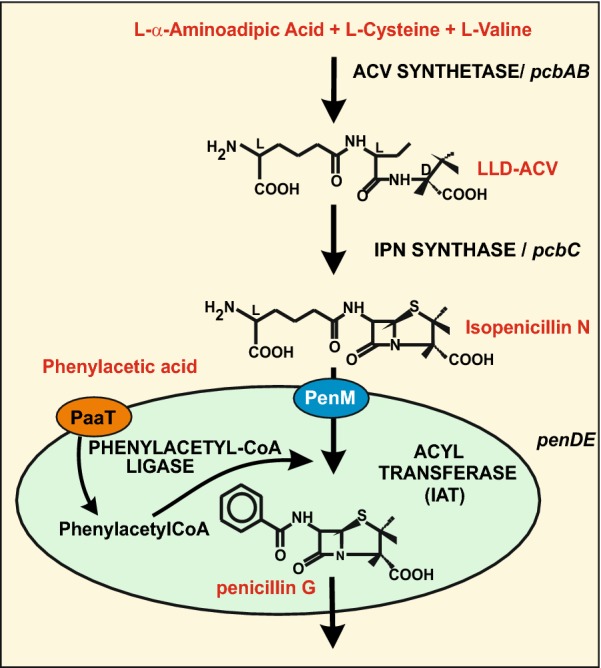
Penicillin biosynthetic pathway. Steps of the penicillin biosynthetic pathway, indicating in red letters the precursors, intermediates and final product. The enzymes/genes are shown at the right side. A peroxisome is shown as a green circle in which the last enzymes of the pathway are included. In the peroxisomal membrane a blue ellipse indicates the PenM protein involved in isopenicillin N transport, and an orange ellipse shows the PaaT protein for phenylacetic acid import into the peroxisomes

The biosynthesis of β-lactam antibiotics has been studied for a few decades and there is a very good understanding of the enzymology, formation of precursors and the regulation of biosynthesis [2, 14, 70]. Recently the molecular mechanisms involved in β-lactam biosynthesis have been reviewed [75] and, therefore, a detailed information on their biosynthetic pathways is not included in this article (Fig. 1).

### Penicillin producing cells in hyphae of *P. chrysogenum*

Growth of *P. chrysogenum* occurs by rapid elongation of hypha tips, as occurs also in other filamentous fungi [43, 99]. Early studies on the identification of penicillin producing active cells showed that penicillin is synthesized in cells in a subapical region of *Penicillium* hyphae [84]; there is no production of penicillin in the actively growing hyphal tip cells. Similarly, production of the aflatoxins by *Aspergillus parasiticus* has been reported to be associated with specific cells in the filamentous hyphae [60, 92]. Electron microscopy studies of the hyphae of *P. chrysogenum* in a high penicillin producing mutant as compared to a low producer strain showed that the active penicillin producing cells in the subapical region contain a high number of large peroxisomes and are partially vacuolated but in the old parts of the hyphae the cells are more highly vacuolated and do not produce penicillin [58]. The subapical penicillin producing cells contain isopenicillin *N* acyltransferase (IAT) in peroxisomes as shown by immunoelectron microscopy [32, 81, 82]. All the available evidence suggests that the entire process of penicillin compartmentalization and transport between intracellular organelles and the cell membrane/cell wall is highly structured and has a precise spatial organization [58]. This article describes the present status of knowledge of the localization and organization of different penicillin and cephalosporin biosynthetic steps and the traffic systems between the different organelles including the controversial final penicillin secretion step.

## Origin of the penicillin precursors in vacuoles and localization of the ACV synthetase

The amino acid precursors of penicillin, namely l-α-aminoadipic acid, l-cysteine and l-valine, derive either from the cytoplasm or from the vacuoles. Lendenfeld et al. [61] provided evidence indicating that the l-α aminoadipic acid comes from vacuoles where it is stored. l-α-aminoadipic acid is a non-proteinogenic amino acid that is an intermediate of the lysine biosynthetic pathway, and it is likely that high cytosolic concentrations may be toxic, acting as an antimetabolite of glutamic acid or aspartic acid in protein synthesis. Therefore, its sequestration in vacuoles may be considered as a safety mechanism to avoid its toxicity to the cells (Table 1). The storage of some amino acids in vacuoles [53, 54] is supported by recent evidence of the characterization of an MFS transport located in the vacuole membrane, named PenV (for vacuole) identified in *P. chrysogenum* [27], see “Compartmentalization of the last two enzymes of penicillin biosynthesis in peroxisomes” section). Mutants altered in the *penV* gene showed a significant decrease in the synthesis of the tripeptide LLD-α-aminoadipyl-cysteinyl-valine (ACV) supporting the conclusion that at least some of these amino acids come from vacuoles. This tripeptide is formed by the multienzyme ACV synthetase that has been characterized in several β-lactam producing organisms [22, 69]. There have been some discrepancies in the localization of the ACV synthetase that forms this tripeptide. Lendenfeld et al. [61], based on the results of electrophoretic mobility of this large protein suggested that this enzyme is loosely attached to vacuoles although they do not conclude whether it was located in the outer or inner side of the vacuole membrane. The loose association of the ACV synthetase with the vacuole membrane was supported by experiments showing that the ACV synthetase activity increases by treatment of disrupted cells with Triton X, or by sonication [94]. Later, van der Lende et al. [107] using both subcellular fractionation and immunoblotting studies, concluded that the ACV synthetase is located in the cytosol without significant attachment to any membrane system.

**Table 1:** Physiological benefits of compartmentalization of β-lactam biosynthetic enzymes

Physiological mechanisms.	Examples of benefitial effects
Sequestration of toxic intermediates or final products	Detoxification of phenylacetic or phenoxyacetic acid by transport into peroxisomes
Channeling of precursors or substrates for β-lactams biosynthesis away from primary metabolism	Storage of α-aminoadipic acid in vacuoles, away from the lysine biosynthetic pathway
Sequestration of intermediates for the temporal sequential formation of intermediates to final products	Temporal conversion of isopenicillin N into benzylpenicillin
Metabolic coupling of biosynthetic reactions and transfer of intermediates between co-localized enzymes	Putative coupling of ACVS and IPNS in the cytosol. Coupling of Phenylacetyl-CoA ligase and IPN acyl transferase Coupling of fatty acids catabolic and modifying enzymes
Localization in organelles having optimal pH or physiological conditions for the biosynthetic enzymes	IAT optimal activity at the pH values at peroxisomes Preservation of the thiol (-SH group) of the tripeptide under reduced redox conditions at the cytosol
Co-localization of enzymes in the membrane, or near the membrane of organelles for joint inclusion in transport vesicles for secretion	Protein assembly that includes VP16 and other proteins of the recognition/teethering membrane complex
Accumulation in vacuoles of proteins and intermediates to be degraded and recycled for biosynthesis of other metabolites	Colocalization of proteases and hydrolases in the vacuoles for recycling cellular materials
Formation of protein secretion complexes	Complexes facilitating secretion of secondary metabolites

## Co-localization of ACV synthetase and IPN synthase

The finding of both ACV synthetase and IPN synthase activities in soluble fractions obtained from *P. chrysogenum* entire cells or from *Penicillium* protoplasts, suggests that these two enzymes may be associated in the cytosol thus favouring metabolic channeling of intermediates as occurs in other examples of metabolic channeling [46] (Table 1). The co-localization of ACV synthetase and IPN synthase may provide an efficient kinetics for the transfer of the ACV released from the ACV synthetase to the IPN synthase, avoiding the loss of the ACV to the cytoplasm and then its release to the external medium where is known to accumulate at least in low producing strains [66]. Since the ACV synthetase is a very large protein, it provides a scaffold for the interaction of ancillary proteins thus stabilizing the ACV synthetase-IPN synthase complex. However, protein–protein interaction studies are required to support this hypothesis. Recently, Kurzatkowski and Kuczerowska [58] using immunodetection of IPN synthase provided evidence indicating that this enzyme is indeed located in the cytoplasm but it is not uniformly distributed; rather, it localizes in a few specific subcellular sites in the cytosol; namely the IPN synthase is associated with the endoplasmic reticulum and strongly concentrated around the peroxisomes and near the vacuole tonoplasts. In addition, a surprising observation of these authors revealed that some IPN synthase is present in tube-like structures in the cell wall. The non-homogeneous distribution of IPN synthase in *P. chrysogenum* is intriguing in view of the proposal that biosynthetic enzymes of some secondary metabolites are translocated by small vesicles (e.g. cytoplasm to vacuole transporter vesicles) [92]. The observed signal and the immunodetection may be related to IPN synthase in traffic vesicles (see “Inactivation of pexophagy increases cephalosporin production” section below).

**Fig. 2:**
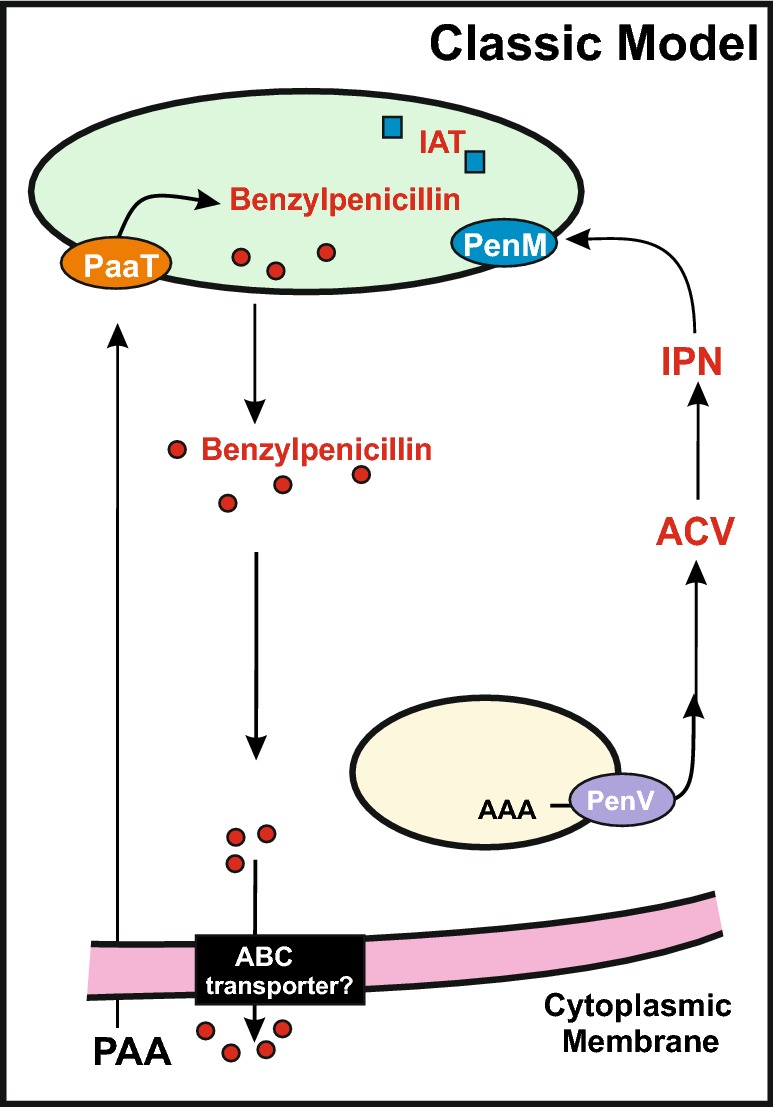
Classic model of compartmentalization of the penicillin biosynthesis pathway in *Penicillium chrysogenum*. Both IPN and PAA are transported into peroxisomes (large green ellipse) by the PenM (blue ellipse) and PaaT (orange ellipse) transporters. The release of the α-aminoadipic acid from IPN (forming 6-APA) and the acylation mediated by the IAT (blue squares) reaction forming benzylpenicillin occurs in peroxisomes. The benzylpenicillin produced is transported to the cytosol and finally secreted to the external medium by a hypothetical ABC transporter (black rectangle) which has not been found so far. A vacuole is shown as a yellow ellipse exporting α-aminoadipic acid to the cytosol through the PenV transporter (purple ellipse)

Finally, the localization of part of the IPN synthase around the peroxisomes is in good agreement with the well-known localization of the enzyme for the next step of the pathway, isopenicillin *N* acyl transferase that converts IPN to benzylpenicillin in peroxisomes (Fig. 2).

### Fate of the isopenicillin N formed

It is interesting that a significant part of the IPN is secreted to the extracellular medium during the early stages of penicillin producing cultures. In vivo studies using genetically tailored strains of *P. chrysogenum* showed that extracellular IPN is not significantly converted in vivo into benzylpenicillin due to its poor uptake, at difference of what occurs with 6-APA (see below) [31]. This finding suggests that the IPN is an important secreted metabolite (extrollite) itself, and its temporally and spatially different secretion constitutes a distinct process from that of conversion to benzylpenicillin by a more complex pathway through peroxisomes. In other words, the penicillin pathway is, indeed, a route for the formation of two different antibiotics IPN and benzylpenicillin that may have different biological functions in nature. At this time it is unclear how the isopenicillin N is secreted to the extracellular medium although it is well stablished that is not taken again by the cells [32].

## Compartmentalization of the last two enzymes of penicillin biosynthesis in peroxisomes

In the last decades a large amount of information has accumulated demonstrating that different enzymes for the biosynthesis of secondary metabolites are located in microbodies and particularly in peroxisomes, i.e. microbodies containing catalase and other enzymes sets [12, 48, 52, 72, 81]. The last two enzymes of the penicillin pathway are involved in the activation of the phenylacetic acid or other aromatic acids to their CoA activated forms such as phenylacetyl-CoA, or phenoxymethyl-CoA. The last enzyme of the penicillin pathway uses phenylacetyl-CoA or phenylacetyl-glutathione (in vitro), as phenyl acetyl donors, to exchange the side chain of IPN to form benzylpenicillin (penicillin G), or phenoxymetyl-CoA to form phenoxymethylpenicillin (penicillin V) although the relevance in vivo of phenylacetyl-glutathione has not been studied in detail [2–4]. The phenylacetyl-CoA ligase (PhlA) was shown to contain the *pts1* targeting sequence by Lamas-Maceiras et al. [59] and its location in peroxisomes was confirmed by Kiel et al. [51] using purified peroxisomes. Other fatty acid acyl CoA ligases also occur in peroxisomes, such as enzymes activating adipic acid, octanoic acid and even longer fatty acids [55, 110, 115]. These last enzymes activate the biosynthesis of penicillins containing linear fatty acid side chains but do not appear to contribute significantly to benzylpenicillin formation [73, 115]. Additional putative p-coumaroyl-CoA ligases, similar to phenylacetyl-CoA ligase have been also found in peroxisomes [73, 106]. All these enzymes contain *pts1* peroxisome targeting signals.

The peroxisomal location of the last enzyme of the penicillin pathway, isopenicillin *N* acyltransferase (IAT), has received much attention since the original work of Müller et al. [81]. In agreement with the peroxisomal localization of IAT there are several studies that indicate that proliferation of peroxisomes enhances IAT activity and therefore penicillin biosynthesis [49, 50]. For example, the biosynthesis of penicillin increases by overexpression of the Pex11 gene, encoding a peroxisomal membrane protein [49]. It has been clearly shown that this enzyme located in peroxisomes is required for the biosynthesis of penicillin; removal of the *pts1* targeting sequence results in the mislocalization of IAT in the cytosol what results in lack of penicillin biosynthesis [78, 82]. However, in *Aspergillus nidulans* a similar mutant still produces traces of penicillin even in complete absence of peroxisomes [98]. The production of penicillin in the IAT deficient mutants in *A. nidulans* is due to an alternative cytoplasmic acyltransferase (AatB) [97] but *P. chrysogenum* mutants in the orthologous *ial* gene, encoding a cytoplasmic IAT-like protein, are not able to synthesize benzylpenicillin [34]. The evolutionary relationship between the IAT and the cytoplasmic acyltransferase has been discussed by García-Estrada et al. [34] and Spröte et al. [97].

Due to the localization of the early biosynthetic enzymes in the cytosol, in contrast to the peroxisomal residence of the phenylacetyl-CoA ligase and IAT, the progress of reactions in the penicillin biosynthesis route requires transport of the side chain precursors and cofactors needed for the final steps of penicillin biosynthesis. There is information on the transport into peroxisomes of fatty acids and cofactors [7, 74] but, until recently, there was very little information on the import of small aromatic or heterocyclic pathway intermediates into peroxisomes [28, 29].

## Transport of IPN into peroxisomes

A major facilitator superfamily (MFS) transporter was found in *P. chrysogenum,* that is similar to a membrane protein located in microbodies of *A. chrysogenum* [29]. The *P. chrysogenum* protein named *penM* (for microbodies) is encoded by Pc21g09220 gene. PenM has 12 transmembrane spanning domains (MSD) and a size of 508 amino acids [29].

Interestingly, the PenM MFS protein contains a Pex19 recognition sequence. The Pex19 protein is a peroxisomal protein that recruits other proteins to be incorporated in the peroxisomal membrane [91].

A *penM* overexpressing strain produces increased levels of penicillin ranging from 169 to 236% with respect to the parental strain *P. chrysogenum* Wis54-1255. These results clearly indicate that transport of isopenicillin N into peroxisomes is rate limiting for penicillin biosynthesis in complex production medium. Several mutants silenced in the expression of the *penM* gene of *P. chrysogenum* Wis54-1255 showed reduced benzylpenicillin production, particularly strain SilM-35 that showed 90% reduction. The isopenicillin *N* acyl transferase activity of the *penM*-silenced transformants is still normal, even in transformant SilM-35, as shown by immunoblotting assays of IAT and in vitro determination of its enzyme activity. These results demonstrate that the *penM* silenced strain is deficient in transport of intermediates but the mutation does not affect the normal incorporation of IAT into peroxisomes.

Confocal microscopy studies using labelled PenM-DsRed protein and the peroxisomal control marker protein EFG-SKL, showed that both proteins co-localize in peroxisomes. This evidence supports the conclusion that PenM is a “bona fide” peroxisomal membrane protein. Experiments with the SilM-35 silenced transformant using increasing concentrations of phenylacetic acid and/or 6-APA show that in this strain its low penicillin biosynthesis is independent of the concentration of phenylacetic acid provided, suggesting that the transport of phenylacetic acid is not mediated by the PenM transporter but is performed by a different phenylacetic acid carrier (see below). On the other hand, interesting results were obtained when increasing amounts of 6-APA were provided to this silent mutant.

### The isopenicillin *N* acyl transferase is easily accessible to external 6-APA

**Fig. 3:**
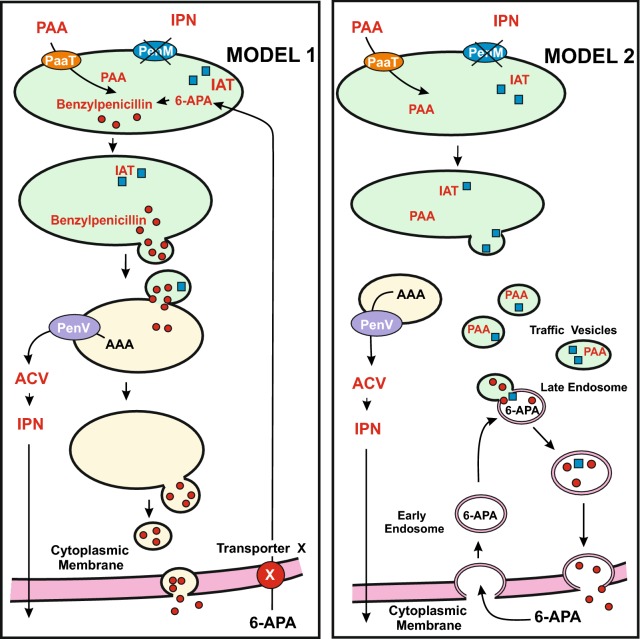
Models for the conversion of external 6-APA into benzylpenicillin and its secretion in *penM* mutants blocked in IPN import in peroxisomes. Peroxisomes are shown as green ellipses. Vacuoles are shown as yellow ellipses. *Model 1* (left side). Conversion of 6-APA into benzylpenicillin in peroxisomes. 6-APA may be introduced in the cells through an unknown cell membrane transporter X, indicated by a red circle. The transporter PenV, indicated with a purple ellipse, exports α-aminoadipic acid from vacuoles to the cytosol. The IAT protein is shown as small blue squares. *Model 2* (right side). Conversion of 6-APA into benzylpenicillin in cytosolic traffic vesicles where the acylation of 6-APA may occur. This implies that IAT is transferred to traffic vesicles. In this model 6-APA is introduced by transporters or endocytosis and is translocated by early endosomes to traffic vesicles. Secretion of benzylpenicillin through the cell membrane/cell wall occurs by fusion with the cell membrane (see text for additional information). For simplicity the early steps of the pathway (biosynthesis of ACV and IPN) are not shown

As indicate above the SilM-35 silenced mutant, deficient in the PenM transporter, is unable to synthesize benzylpenicillin even in a medium supplied with phenylacetic acid. Surprisingly, this mutant produced normal amounts of benzylpenicillin when 6-APA was supplied extracellularly to the cells. [29]. The formation in vivo of benzylpenicillin in cultures supplemented with 6-APA was linearly dependent on the amount of 6-APA supplied to the cells. This result suggests that 6-APA has easy access to the IPN acyltransferase in the peroxisomes or, alternatively it is possible that the IAT get in contact with the 6-APA at the cell membrane or in endosomes or traffic vesicles (Fig. 3). It is also possible that the 6-APA is internalized by endosomes and transported by vesicles into vacuoles thus bypassing the need of IPN in peroxisomes. The 6-APA-dependent formation in vivo of benzylpenicillin in cultures confirms the previous finding of García-Estrada et al. [31] who observed efficient conversion of 6-APA in penicillin using intact cells of tailored *P. chrysogenum* strains that lack the *pcbAB* and *pcbC* genes and contains only the IAT encoding gene (*penDE*). The efficient mechanism of internalization of 6-APA has not been studied, although it may involve either an unknown transporter or the internalization through formation of endosomes, as observed in *A. nidulans* for other organic molecules such as the FM4-64 dye [1, 85]. The FM4-64 dye is internalized in early endosomes formed in the subapical cells by an endocytosis process and move bidirectionally on microtubule tracks [43, 85]. Endosomes containing different cargo molecules fuse with vesicle transport systems that serve as carriers in secretion of secondary metabolites [16, 17, 64] (see below “Inactivation of pexophagy increases cephalosporin production” section). In summary, the SilM-35 mutant lacking the *penM* gene is still able to convert 6-APA to benzylpenicillin and secret it. This finding and the information provided by Chanda et al. [18] is useful to visualize a new model for the secretion of benzylpenicillin after synthesis of this antibiotic in the cells (Fig. 3; see “Inactivation of pexophagy increases cephalosporin production” section).

## Toxicity, detoxification and transport into peroxisomes of phenylacetic acid

The benzylpenicillin side chain precursor phenylacetic acid (PAA) is toxic to *P. chrysogenum* and other fungi [25, 59], and its undissociated form, PAA, is transported by passive or facilitated diffusion through the plasma membrane [42]. Once inside the cells the phenylacetate molecule releases one proton and therefore acidifies the cytosol [112] as occurs with other weak organic acids. Also, phenylacetic acid and organic acids may disturb the membranes in the cells and disrupt the respiratory chain in mitochondria. To avoid this toxicity the *P. chrysogenum* cells have developed several detoxification mechanisms. The first mechanism involves extrusion of excess phenylacetic acid from the cells. Weber et al. [112] studied the response of the genes encoding the 48 ABC transporters in *P. chrysogenum* and found that one of them, ABC-40 is induced by addition of extracellular phenylacetic acid. The encoded ABC transporter was identified as an exporter of weak acids and is functionally similar to the PDR12 transporter of *Saccharomyces cerevisiae* [112]. A second detoxification mechanism was initially found in *A. nidulans* [79]; this mechanism is weaker in *P. notatum* and is very weak in *P. chrysogenum* [88, 89]. This second mechanism proceeds through hydroxylation of phenylacetic acid by a monooxygenase that results in the initial formation of 2-hydroxyphenylacetic acid that is later further oxidized and degraded through the homogentisic acid pathway to form finally succinic acid and acetyl-CoA [30].

Whereas *P. notatum* is able to grow on phenylacetic acid as carbon source, *P. chrysogenum* is unable to do so due to a mutation in the gene encoding a phenylacetic acid monooxygenase that hydroxylates PAA at carbon-2 [88, 89].

The low or null phenylacetate degrading activity in *P. chrysogenum,* particularly in the improved penicillin producing strains, differs from the well-known high phenylacetate catabolism activity found in *A. nidulans*, that is able to degrade phenylacetic acid using at least two different enzyme systems. One of them encodes the phenylacetate 2-hydroxylase (PAA-hydroxylase) [79] and a second one degrades phenylacetic acid by hydroxylating at carbon-3, and also at carbon-4 forming 3,4 dihidroxyphenylacetate that is later fully degraded [30]. Recently, Jami et al. [45] in proteomic studies of *P. chrysogenum* have found two additional PAA monooxygenase homologs that contain more than 60% amino acid identity to the PahA hydroxylase reported by Rodríguez-Sáiz et al. [88].

The third phenylacetate detoxifying pathway involves the transport of phenylacetic acid in peroxisomes and its conversion to phenylacetyl-CoA that is subsequently used for penicillin biosynthesis.

The transport of phenylacetic acid into peroxisomes has remained obscure for many years, but recently Fernández-Aguado et al. [28] identified an MFS transporter, named PaaT, that affects penicillin biosynthesis and is involved in the transport of PAA into peroxisomes; the same gene has been cloned by Yang et al. [113] from a different *P. chrysogenum* strain. This MFS transporter encodes a protein of 548 amino acids that contains 12 transmembrane spanners. Mutants silenced in the *paaT* gene of *P. chrysogenum* showed greatly reduced biosynthesis of penicillin. Normal biosynthesis of penicillin was restored by complementation with a wild type *paaT* allele. Confocal fluorescent microscopy using both a *paaT*-DsRed red labelled protein and the peroxisome targeted EGFP-SKL green fluorescent protein as marker for peroxisome location demonstrated that the PaaT transporter is located in the peroxisomal membrane [28]. Expression of the *paaT* gene is strongly increased by addition of phenylacetic acid to the medium (22.7-fold in *P. chrysogenum* Wis54-1255 and 170-fold in an industrial strain) [105]. This impressive high degree of phenylacetic acid induction of *paaT*-mRNA and subsequent stimulation of penicillin biosynthesis indicates that this gene is involved in penicillin production in response to phenylacetic acid. Overexpression of the *paaT* gene resulted in overproduction of penicillin (40 to 100%) in different transformants, and also lead to higher resistance to phenylacetic acid, whereas the silenced transformants were more sensitive to phenylacetic acid. These results clearly indicated that import of phenylacetic in peroxisomes decreases the intracellular phenylacetic acid in the cytosol and its toxicity. Interestingly, genes homologous to *paaT* are present in the genome of *A. nidulans,* A. *oryzae* (both penicillin producers) and also of *Aspergillus clavatus* and other fungi that are not known to produce penicillin, suggesting that this transporter is common in fungi for the import of organic acids similar to phenylacetic acid, such as coumaric acid, cinnamic acid, salicylic acid or caffeic acids that are precursors for the biosynthesis of flavonoids and similar compounds [23, 95].

In addition to 12 MSD, the PaaT protein also has a Pex19-interacting motif [91]. In summary, the PaaT transporter forms part of a mechanism of detoxification of PAA and related organic acids that is common to several filamentous fungi.

## The peroxisomal IAT is a NTN hydrolase with five related enzyme activities: maturation by self-processing

Initial work on the purification of the IAT showed that it has not only isopenicillin N acyl transferase activity (IAT) but also a strong 6-APA acyl transferase activity that uses 6-APA as substrate instead of isopenicillin N for the acylation [4]. Three other activities were also found in the same protein, namely the isopenicillin N amido hydrolase (releasing α-aminoadipic acid), penicillin G and penicillin V transacylase activity, and hydrolysis of benzylpenicillin to 6-APA (releasing 6-APA) [5]. The five activities are lost in the *penDE*-null mutant and are regained when the mutant is complemented with the *penDE* gene [5, 11]. The *penDE* gene encodes the pro-IAT, a N terminal nucleophile (NTN) hydrolase, which is self-processed by cleavage between gly^102^ and cys^103^ forming the α and β subunits, probably in the peroxisomes [8, 101]. The molecular basis of the processing has been rewieved elsewhere [75]. Unprocessed IAT molecules still are targeted to the peroxisomes and localized in these organelles [32]; it seems that maturation of the pro-IAT occurs in peroxisomes, where the pH is optimal for the processing as well as for IPN cleavage since both catalytic processes share the same molecular mechanisms (Table 1) [75].

The IAT proteins of *P. chrysogenum* and *A. nidulans* differ in their ability to split the α and β subunits. The *P. chrysogenum* small α subunit is easily separated from the β subunit during the cell disruption and purification process while the two subunits of the *A. nidulans* enzyme remain at least partially attached together [26]. The gly^102^ and cys^103^ residues are maintained in both IATs; other important residues are different [80] and this may explain the distinct behaviour of both IATs after the initial autoproteolytic cleavage.

### Alternative routes of penicillin secretion: Lack of correlation of pexophagy with β-lactams secretion

*Penicillium chrysogenum* is able to secret very large amounts of penicillin under optimal conditions for penicillin production. However, in spite of the interest in understanding the molecular mechanisms of penicillin secretion there has been little progress in this field. Following an early report of the impact of an ABC transporter of *A. nidulans* in penicillin production [6] it was proposed that penicillin might be secreted might an ABC type transporter. This *A. nidulans* gene affects penicillin production but mutation of this gene does not prevent penicillin secretion. Studies at the DSM research laboratories of the 48 ABC transporters known in *P. chrysogenum* indicate that none of these transporters is responsible for penicillin secretion although one of them, ABC-40 was involved in active phenylacetic acid secretion and might impact penicillin production [112].

Several pathways for secretion of penicillin in *P. chrysogenum* have been proposed [73] but the real mechanism in the high producing strains is still obscure (Fig. 3). One possible mechanism involves macrophagy of organelles forming the autophagosome that is later fused to the vacuoles therefore releasing its content into the vacuolar lumen. This mechanism has been studied in yeasts but is still poorly known in filamentous fungi [86, 109, 114]. The autophagy process affects different organelles, in particular the autophagy of peroxisomes is named pexophagy. If peroxisomes are engulfed by a pexophagy mechanism the penicillin and the biosynthetic enzymes will be discharged into the vacuoles [58]. Using electron transmision macroscopy, Kurzatkowski and Kuczerowska [58] have also observed the accumulation of debris material inside the vacuole tonoplasts and budding structures in the vacuoles that might be involve in exocytosis. No similar accumulation of debris material has been observed in wild type low penicillin producing strains [58]. This observation may be consistent with mechanisms in which the vacuoles are fused to the cellular membrane and the vacuoles content is extruded to the extracellular medium.

The involvement of pexophagy in recycling cellular material in *P. chrysogenum* was demonstrated by following the label of GFP-SKL markers from peroxisomes to the vacuole lumen [12]. This was particularly relevant in cells after 78 h, a phase in which the cells start an organelles rearrangement that leads to cell lysis. Consisting with this finding was the observation that autophagy deficient mutants lacked the transfer of GFP-SKL marker to the vacuoles. However, recent evidence indicates clearly that the pexophagy does not correlates with penicillin biosynthesis. Noteworthy, the pexophagy-deficient strains show a significant increase of penicillin production under penicillin inducing conditions [12] The increasing levels of penicillin production correlates with an enhanced number of peroxisomes in the subapical hyphal cells indicating that the mutation in autophagy preserves the peroxisomes from degradation. This also correlates with increasing levels of penicillin biosynthetic enzymes. In conclusion, the available evidence suggests that indeed the penicillin secretion mechanism from peroxisomes does not relay on the pexophagy mechanism since this degrades the peroxisomes damaging penicillin biosynthesis. Support for this hypothesis has been obtained recently in the cephalosporin producer *A. chrysogenum* [19, 62, 65] (see below “Cephalosporin export: the MFS transporter *cefT* increases cephalosporin secretion” section).

## Compartmentalization of enzymes involved in cephalosporin biosynthesis in *Acremonium chrysogenum*

**Fig. 4:**
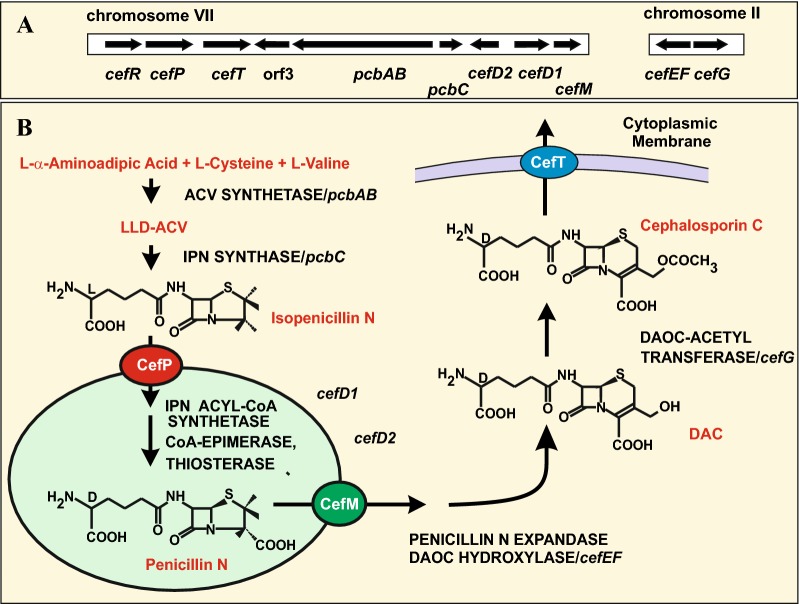
Gene cluster and compartmentalization of the cephalosporin biosynthesis pathway. **a** Genes located in chromosome VII encoding transporters and enzymes for the early steps of the pathway are shown at the left side. Genes for late enzymes, located in chromosome II are shown at the right side. **b** Steps of the cephalosporin C pathway, indicating in red letters the precursors, intermediates and final product. The enzymes/genes are shown at the right side. Peroxisomes are shown as a green circle in which the enzymes involved in isopenicillin N epimerization are included. In the peroxisomal membrane a red ellipse indicates the CefP protein involved in isopenicillin N import, and an orange ellipse shows the CefM protein for penicillin N export. The cytoplasmic membrane is highlighted in purple and the CefT protein, for cephalosporin C secretion, in a blue ellipse

Cephalosporin C (CPC) produced by *A. chrysogenum* has a wide spectrum of antibacterial activity against Gam-positive and Gram-negative bacteria. The first two steps of cephalosporin biosynthesis are identical to those involved in penicillin formation in *Penicillium* and *Aspergillus* (Fig. 4b) [38, 71], namely the formation of the tripeptide α-aminoadipyl-cysteinyl valine and cyclization of this lineal tripeptide to form isopenicillin N [93]. Formation of the tripeptide ACV is catalysed by the multienzyme ACV synthetase, that has been well characterized [10, 38]. In addition, a long pathway converts isopenicillin N into CPC, a conversion that is mediated by several enzymes and does not occurs in *Penicillium* or *Aspergillus*. First, the isopenicillin N is epimerized to penicillin N by the combined action of the isopenicillin N-CoA ligase and the isopenicillin N-CoA epimerase encoded by genes *cefD1* and *cefD2* [71, 103]. The penicillin N is converted first into deacetoxycephalosporin C (DAOC) and then to deacetylcephalosporin C (DAC) by the bifunctional enzyme DAOC synthase/hydroxylase, so called expandase, that is encoded by a single gene, *cefEF* in the *A. chrysogenum* genome [24]. In the last step of the CPC pathway the DAC is acetylated to form cephalosporin C [39, 108]. The genes encoding the enzymes of the CPC biosynthetic pathway are organized into two different clusters. The early genes of the CPC cluster are located in chromosome VII that contains the *pcbC pcbAB, cefD1, cefD2* and 3 other genes (Fig. 3a) involved in intracellular transport and secretion [40, 100, 102, 104].

*A. chrysogenum* has a second cluster, located in chromosome II, or in chromosome I in the industrial strain *A. chrysogenum* C10, that contains the *cefEF* and the *cefG* genes, encoding the two last enzymes of the CPC pathway [39]. There is little information on the subcellular localization of the CPC biosynthetic enzymes. The first two enzymes, ACV synthetase and IPN synthase, are believed to be cytosolic enzymes, as occurs also in *P. chrysogenum.* However, the two enzymes involved in the epimerization of isopenicillin N to penicillin N, encoded by *cefD1* and *cefD2*. are located in peroxisomes as concluded from two different observations. First, the IPN-acyl-CoA ligase contains a *pts1* peroxisomal targeting sequence and the IPN acyl-CoA epimerase contains both a *pts1* and a *pts2* targeting sequences. Second, results from the peroxisomal proteome indicated that enzymes, homologous to CefD1 and CefD2, are located in peroxisomes also in *P. chrysogenum* [51]. The two last enzymes of the CPC pathway, namely the DAOC synthase/hydroxylase and the DAC acetyl transferase, are believed to be cytosolic although there is no experimental confirmation of this localization. The compartmentalization of the IPN CoA ligase and IPN CoA-epimerase suggests that intracellular transport of intermediates in and out of peroxisomes is critical for the logistics of cephalosporin biosynthesis. Unless these intermediates are properly transported the CPC pathway will be blocked and only isopenicillin N would be produced.

Three genes located in the early CPC cluster encode MFS transporters, two of them CefM and CefP corresponds to transporters of intermediates in and out of peroxisomes [100, 104]. Both the CefM and CefP protein contain Pex19 interacting motifs [91] suggesting that these MFS transporters are recruited by Pex19 and integrated in the peroxisome membrane.

Genetic analysis of the first transporter CefM indicates that this protein belongs to family 3 (drug/proton efflux proteins) of MFS transporters. Disruption of the *cefM* gene prevented CPC biosynthesis and resulted in accumulation of penicillin N, indicating that this transporter is involved in the secretion of penicillin N from the peroxisomes and its conversion in DAC. The two last enzymes of the CPC pathway were not affected in the *cefM*-disrupted mutant. Fluorescensce microscopy studies evidence that the CefM protein is located in peroxisomal membranes [100].

The second transporter, CefP is also a MFS protein of 12-transmembrane domains (eleven + one poorly conserved) that is located in the peroxisomal membrane as shown by using a CefP-DsRed functional fusion protein [104]. Disruption of the *cefP* gene completely blocked CPC biosynthesis but interestingly this mutant accumulates IPN demonstrating that this MFS transporter is involved in the introduction of IPN into peroxisomes. The size of CefM and CefP and their amino acid sequences are different and this indicates clearly that they play different roles in the introduction of intermediates in peroxisomes and the final conversion of these intermediates to CPC.

## Cephalosporin export: the MFS transporter *cefT* increases cephalosporin secretion

In 2002 a third MFS transporter encoding gene, *cefT,* was located in the early CPC gene cluster [103]. This gene encodes a protein of the MFS class containing 12 MSDs and all the five characteristic motifs of the drug/H^+^ antiporters. The *cefT* gene is located downstream of the *pcbAB* gene in the CPC early cluster (Fig. 4A). A gene orthologous to *cefT* does not exists in *P. chrysogenum* or *A. nidulans.* Disruption of *cefT* did not prevented CPC production and has no effect on growth of *A. chrysogenum.* However, overexpression of this gene resulted in a 90% increase of CPC formation. Expression of a truncated version of *cefT* did not produced the same CPC stimulatory effect, supporting that the CPC overproducing effect is, indeed, due to enhanced expression of the complete *cefT* transporter. Since disruption of *cefT* did not prevented CPC production it is concluded that CPC is secreted by redundant transporter systems, in other words, in addition of *cefT* there are alternative CPC extrusion systems.

Later Nijland et al. [83] expressed the *cefT* gene of *A. chrysogenum* in a modified *P. chrysogenum* strain that produced adipoyl-7-amino-3-carbamoyloxymethyl-3-cephem-4-carboxylic acid (ad7-ACCCA), a hybrid β-lactam antibiotic. Expression in *P. chrysogenum* of the *cefT* gene of *A. chrysogenum* resulted in an almost two-fold increase of ad7-ACCCA production in this host *P. chrysogenum* strain. Studying expression of a CefT-GFP fusion protein these authors observe that the fluorescence was located both in the vacuoles and in the cell-membrane, a phenomenon that is not uncommon when a membrane gene is overexpressed. In conclusion, it seems that secretion of CPC in *A. chrysogenum* and ad7-ACCCA in *P. chrysogenum* is mediated at least in part by the MFS transporter CefT.

## Inactivation of pexophagy increases cephalosporin production

As indicated above pexophagy does not correlate with penicillin production in *P. chrysogenum.*

Ubiquitin-like proteins (named ATG) are involved in the formation of the pre-autophagosome and the maduration of the phagosome and its incorporation to the vacuoles.

Two of the *A. chrysogenum* ATG encoding genes were cloned and disrupted. Disruption of the gene encoding one of the ATG proteins, AC-ATG1, resulted in an increase of cephalosporin production but also affected considerably growth of the mutant strain [111]. More recently, Chen et al. [19] studied the AC-ATG12 protein and its role in pexophagy. In fungi the autophagosome protein ATG12 plays a key role in the fusion of the ATG8 protein with phosphatidylethanolamine. Disruption of the gene encoding this protein in *A. chrysogenum* resulted in a two-fold increase in cephalosporin C production, in agreement with the previous observation with the ATG1 protein [111]. The AC-ATG12 mutants were also impaired in sporulation but the mutation does not affect vegetative growth, therefore supporting good cephalosporin production. Interestingly, the AC-ATG12 mutant overexpressed all the cephalosporin biosynthetic genes and this is probably indirectly related to the preservation of functional peroxisomes, although further studies are required to confirm this fact. The increase expression of all cephalosporin genes, determined by RT-PCR, is really intriguing and has not been explained so far. It seems that the transcription of these genes is negatively affected by the onset of autophagy and this does not occur in the ATG12 mutant that lacks pexophagy. Since the seven genes for cephalosporin biosynthesis are transcribed from separated promoters [38, 39], the coordinated regulation of all the genes indicates that a master transcriptional factor or a regulatory system such as LaeA/Velvet complex [13, 56, 76] is probably involved in the coregulation of these promoters.

## The V subcellular fraction: vesicles versus vacuoles in the way out of secondary metabolites from the cells

There is increasing evidence that in different fungi multienzymatic protein complexes are involved in the biosynthesis of secondary metabolites facilitating metabolic channelling [46]. Indeed, different enzymes involved in the biosynthesis of secondary metabolites are located in membrane surrounded organelles such as peroxisomes, vacuoles or traffic vesicles/endosomes [63]. Important advances on the elucidation of intracellular traffic involved in the biosynthesis of aflatoxin in *A. parasiticus* have been made by Chanda et al. [16], Roze et al. [92] and Linz et al. [64]. Chanda et al. [16, 17] isolated a highly purified vesicles fraction, so called V-fraction, that is able to convert sterigmatocystin into aflatoxin B1. This fraction includes transport vesicles and endosomes, and contained the middle and late aflatoxin biosynthetic enzymes. The final products aflatoxins were also located into these vesicles/endosome fraction that were designated “aflatoxisomes”.

Traffic vesicles are double layer membrane structures that transport cargo proteins between different subcellular locations. They may fuse directly with vacuoles or alternatively with endosomes and other vesicles to form multivesicular structures, and finally merge with the vacuoles. The endosomes derive from endocytosis of the cell membrane and are able to internalize and transport extracellular compounds to the vacuoles. These studies provided evidence indicating that inhibition of the fusion of vesicles with vacuoles increases aflatoxin biosynthesis supporting the conclusion that vesicles are the organelles primarily involved in the intracellular transport of aflatoxins [18]. This brings out the question of how aflatoxins are targeted to the cell membrane and secreted if they are not going through the vacuoles. These authors propose that the secretion mechanism involves interaction of aflatoxisomes with the cell membrane and extrusion of the aflatoxin and other cargo material [64, 92].

## Are vesicles and endosomes involved in penicillin secretion in *Penicillium chrysogenum*?

On the light of the findings in *A. parasiticus* transport vesicles and endosomes an interesting question is whether similar mechanisms may play a role in secretion of penicillin in *P. chrysogenum* (Fig. 3). Some information is provided by the results of the localization of penicillin biosynthetic enzymes in microbodies and microbodies associated particles [51]. These authors obtained microbodies preparation by gentle disruption of *P. chrysogenum* protoplasts filtered through glass wood in 1.2 M sorbitol buffer with protease inhibitors. Lised protoplasts were subjected to control centrifugation and later purified in a sucrose gradient. The 30.000xg pellet fraction contains mainly mitochondria, and microbodies. After ultracentrifugation in a sucrose gradient microbodies were separated from mitochondria and immunodetection assays of IAT as marker for microbodies or cytochrome C oxidase as control of mitochondria were made [51]. Further lysis of the purified microbodies revealed that IAT is located in the peroxisomal matrix. Although it is clear that IAT is located in microbodies these results do not exclude that traffic vesicles/endosomes may also be collected in the microbodies fraction. Noteworthy enzymes involved in biosynthesis of secondary metabolites other than penicillin are present in this fraction that is likely to be located in traffic vesicles. Remarkably proteins lacking the *pts1* or *pts2* peroxisomal targeting sequences were also associated with the microbodies fraction [51]. These includes some ribosomal proteins that appear to be closely associated with synthesis of microbody proteins, and interestingly ACV synthetase and IPN synthase which lack *pts* signals. These two enzymes have also been found in the cytosol; therefore, it cannot be excluded that the activity found associated with the peroxisomal faction is due to a contamination. It is important to note that penicillin is not accumulated in the cytosol after been formed in perosixomes but is very efficiently secreted against a hundred-fold concentration gradient during the time of maximal production. Therefore, this suggest that there is an efficient translocation system that perhaps may involve traffic vesicles.

This proposal connects with old results on the formation of extracellular vesicles protruding of hyphal cell (bulges) in *P*. *chrysogenum,* observed by scanning electron microscopy that remained partially attached to the cell wall [67] and at that time, were assumed to be related to penicillin secretion [57]. Biochemical characterization of the content of these vesicles will greatly help to understand the penicillin secretion mechanism.

## Concluding remarks

### Benefits of subcellular compartmentalization and difficulties to improve the β-lactam yield by modifying the spatial organization of biosynthetic enzymes for secondary metabolites

As concluding from all available evidence *P. chrysogenum* and *A. chrysogenum* have developed elaborated systems of compartmentalization of penicillin or cephalosporin biosynthetic enzymes. The compartmentalization of the enzymes requires an intracellular traffic of precursors and intermediates to allow antibiotic biosynthesis, and finally an export system mediated by either MSF or ABC transporters, or alternative antibiotic extrusion systems. The development of this elaborated compartmentalization system and its conservation during centuries implies that this system has beneficial effects for the growth, differentiation and survival of the producer strains under stressing environmental conditions. Several beneficial effects of compartmentalization are known and have been summarized in Table 1. Heterologous expression of the penicillin biosynthetic genes in *S. cerevisiae,* has been achieved [9, 37, 96]. Very low concentrations of penicillin were detected in the *S. cerevisiae* culture broth indicating that the yeast compartmentalization and secretion system is not optimal for biosynthesis of this metabolite. An important question is whether the penicillin production may be improved by targeting the enzymes to specific organelles in which, they may have more adequate physiological conditions. The three penicillin biosynthetic enzymes were individually targeted to peroxisomes in *A. nidulans* [41]. These authors changed the subcellular residence of either IPN synthase or ACV synthetase by targeting these enzymes modified with a *pts1* peroxisomal targeting sequence. Interestingly, localization of the IPN synthase in peroxisomes failed to produce any penicillin. It is not surprising that moving the cytosolic IPN synthase to peroxisomes resulted in the loss of penicillin biosynthesis because this enzyme requires oxygen and cofactors for its activity, particularly iron and ascorbic acid, which may not be available at the adequate concentration in peroxisomes. Since, in this case, the ACV synthetase remains in the cytosol, the pathway would require the transport of the ACV tripeptide into the peroxisomes which has not been demonstrated so far. On the other hand, when the ACV synthetase was targeted to the peroxisomes it was functional resulting in a three-fold increase of penicillin production. The ACV synthetase is a large multienzyme and in addition requires its modification by a cognate phosphopantenthenyl transferase [33] prior to entering in the peroxisomes. It seems that enough amount of the three precursor amino acids are available in peroxisomes. However, the product of the ACV synthetase would needs to be secreted to the cytosol to be converted by the IPN synthase into IPN. When both, ACV synthetase and IPN synthase were targeted to the peroxisomes, in addition to the resident IAT, no penicillin production was obtained despite of the presence of the three enzymes in the peroxisomes [41]. These results show that there are difficulties for the metabolic engineering of the subcellular localization of biosynthetic enzymes. Therefore, more basic information on the different transport mechanisms is needed.

## Data Availability

This is a review article and availability of data is not applicable.

## Abbreviations

NRP: Non-ribosomal peptides
PK: Polyketides
IPN: Isopenicillin N
ACV: LLD-α-aminoadipyl-cysteinyl-valine
6-APA: 6-Aminopenicillanic acid
CPC: Cephalosporin C
ABC: ATP-binding cassettes
MFS: Major facilitator superfamily
MSD: Membrane spanning domain
AAA: α-Aminoadipic acid
IAT: Isopenicillin *N* acyltransferase
*pts1* and *pts2*: Peroxisomal targeting sequences
